# Knowledge domain and trends in acupuncture for stroke research based on bibliometric analysis

**DOI:** 10.3389/fnhum.2025.1544812

**Published:** 2025-03-19

**Authors:** Hongdong Hao, Yifang Xing, Jiashu Chen, Haijun Wang, Aiai Dong, Hai-Xin Liu

**Affiliations:** ^1^College of First Clinical, Shanxi University of Traditional Chinese Medicine, Taiyuan, China; ^2^College of Second Clinical, Shanxi University of Traditional Chinese Medicine, Taiyuan, China; ^3^College of Traditional Chinese Medicine and Food Engineering, Shanxi University of Chinese Medicine, Taiyuan, China

**Keywords:** acupuncture, stroke, bibliometrics analysis, CiteSpace, VOSviewer

## Abstract

This bibliometric analysis comprehensively investigates the literature on acupuncture for stroke rehabilitation, aiming to identify key research hotspots, map the underlying knowledge structure, and examine developmental trends. The central hypothesis posits that acupuncture plays a pivotal role in enhancing neurological and motor function recovery in stroke patients, particularly when combined with complementary therapeutic modalities. A total of 2,217 relevant publications, spanning from database inception through 2024, were selected following stringent data screening and cleaning protocols. Utilizing advanced bibliometric tools such as CiteSpace and VOSviewer, we analyzed publication trends, leading authors, influential institutions, and citation networks. Our findings reveal a consistent and significant increase in research activity, with China emerging as the predominant contributor to this field. The analysis strongly emphasizes neurological recovery, motor function improvement, language rehabilitation, and the integration of acupuncture with other therapeutic strategies. Prominent keywords, including “acupuncture treatment,” “scalp acupuncture,” “electroacupuncture,” and “rehabilitation therapy,” reflect the evolving priorities within this domain. This study provides valuable evidence-based insights to guide future research on acupuncture for stroke rehabilitation, offering a solid framework for experimental investigations. By delineating the knowledge landscape, it contributes to refining research hypotheses and optimizing the clinical application of acupuncture in stroke recovery.

**Systematic review registration:**

https://inplasy.com/, INPLASY202530038.

## 1 Introduction

Stroke, a cerebrovascular disease with high rates of disability and mortality, poses a significant threat to human health (Udo, 2024). It has become the leading cause of death among individuals over the age of 60, accounting for a substantial proportion of deaths in this demographic (Xu et al., 2024). In addition, stroke ranks as the fifth leading cause of death among young and middle-aged individuals aged 15–59, highlighting its broad impact across age groups (Curtin, 2024). Current stroke treatment methods include drug therapy, surgical intervention, and rehabilitation therapy (Wadhwa et al., 2024). However, these approaches have certain drawbacks: Drug therapy may come with bleeding risks and side effects, surgical intervention is high risk and not suitable for all patients, and rehabilitation therapy is time-consuming with varying effects depending on individual differences (Foschi et al., 2024). Acupuncture, an ancient Chinese medical treatment, works by stimulating specific acupoints on the body to regulate the flow of qi and blood through the meridians, aiming to unblock the meridians, harmonize yin and yang, and strengthen the body’s defenses against disease (Gamus and Shoenfeld, 2024; Li et al., 2024). In the medical field, acupuncture is widely used for the treatment and prevention of various diseases, showing significant efficacy in pain management, neurological disorders, digestive system diseases, endocrine disorders, and symptoms related to mental stress. In the field of stroke, the advantages of acupuncture treatment are particularly prominent (Guo et al., 2024; Wei et al., 2024). It can significantly improve patients’ motor function, swallowing function, and cognitive function of the brain, promoting patient recovery (Li et al., 2024). Compared to other treatment methods, acupuncture offers the benefits of simplicity, high safety, and minimal side effects, providing an effective adjunctive treatment option for stroke patients (Kalaoglu et al., 2024).

Bibliometrics is an interdisciplinary field that integrates the core theories of mathematics, statistics, and documentation studies (Petrovich et al., 2024). It applies principles and methods from mathematics and statistics to conduct in-depth and precise quantitative analysis and research on knowledge carriers (Li et al., 2024). In the field of medicine, bibliometrics demonstrates its significant academic value. By analyzing papers and their citation relationships, it can outline the research hotspots, knowledge structures, and overall development trends within the medical field (Yang and Liu, 2024). With the analytical methods of bibliometrics, researchers can accurately identify research directions with high impact, thereby exploring potential research directions for scientific work. In addition, it can reveal potential opportunities for scientific collaboration, promoting academic exchange and cooperation between different research teams (Wang et al., 2024; Wang et al., 2024). CiteSpace and VOSviewer are important tools in the field of scientific visualization in bibliometrics (Zhao et al., 2024). CiteSpace can analyze information such as papers, authors, journals, and keywords in academic fields and display the trends and development of academic research by presenting the structure, patterns, and distribution of scientific knowledge through visualization (Zhao et al., 2024). VOSviewer, characterized by its large amount of information and easy-to-understand graphics, supports data import, co-occurrence analysis, network visualization, heatmaps, clustering analysis, and data export, helping researchers better understand the relationships, trends, and hotspots among literature (Yu et al., 2024).

This article utilizes data visualization software represented by CiteSpace and VOSviewer, based on the Web of Science (WOS) and PubMed databases, to visually analyze publications on acupuncture treatment for stroke. This will provide doctors and researchers with a comprehensive and systematic overview of the current state of stroke research, offering new perspectives and methodologies for studying this field. Furthermore, it will provide an evidence base for the accumulation of academic achievements, helping researchers to objectively assess the status and impact of their own research findings within the field of stroke research (Du et al., 2024; Jiao et al., 2024).

## 2 Methods

### 2.1 Search strategy

A systematic search was conducted in the core collections of the Web of Science (WOS) and PubMed databases to identify relevant papers published between 2020 and 2024. The initial query—((TS = (stroke)) AND TS = (acupuncture)) OR TS = (needle)—retrieved a substantial number of irrelevant documents. To improve specificity, the search strategy was refined as follows: (stroke AND acupuncture) AND (cerebrovascular accident AND acupuncture) AND (stroke AND needling) AND (cerebrovascular accident AND needling) AND (stroke AND acu-mox) AND (cerebrovascular accident AND acu-mox). The results from these searches were consolidated for further bibliometric analysis.

### 2.2 Inclusion criteria

Studies were included if they were relevant to the title, keywords, abstract, or indexed subject terms. The search within the WOS database was limited to publications in English and restricted to the following disciplines: clinical neurology, peripheral vascular disease, neurosciences, general internal medicine, and integrative and complementary medicine. Duplicate records were identified and excluded before analysis.

### 2.3 Exclusion criteria

CiteSpace was used to classify the retrieved literature, and the following exclusion criteria were applied: (1) studies addressing stroke without reference to acupuncture therapy; (2) articles discussing acupuncture therapy without specifically focusing on its application in stroke treatment; and (3) non-peer-reviewed literature, including conference abstracts, conference papers, dissertations, letters, news articles, and similar formats.

### 2.4 Screening and data extraction

Two researchers independently reviewed and screened the retrieved literature based on titles, abstracts, and keywords. Any disagreements were resolved by consulting a third researcher, and in cases where the third researcher expressed a differing opinion, the final decision was reached through a group discussion among all three researchers. Data from the WOS database were exported in “full text” format, with full records and cited references extracted to serve as the data source for bibliometric analysis using tools such as CiteSpace and VOSviewer.

## 3 Visualization of literature spatiotemporal results

### 3.1 Literature screening

**Figure 1:**
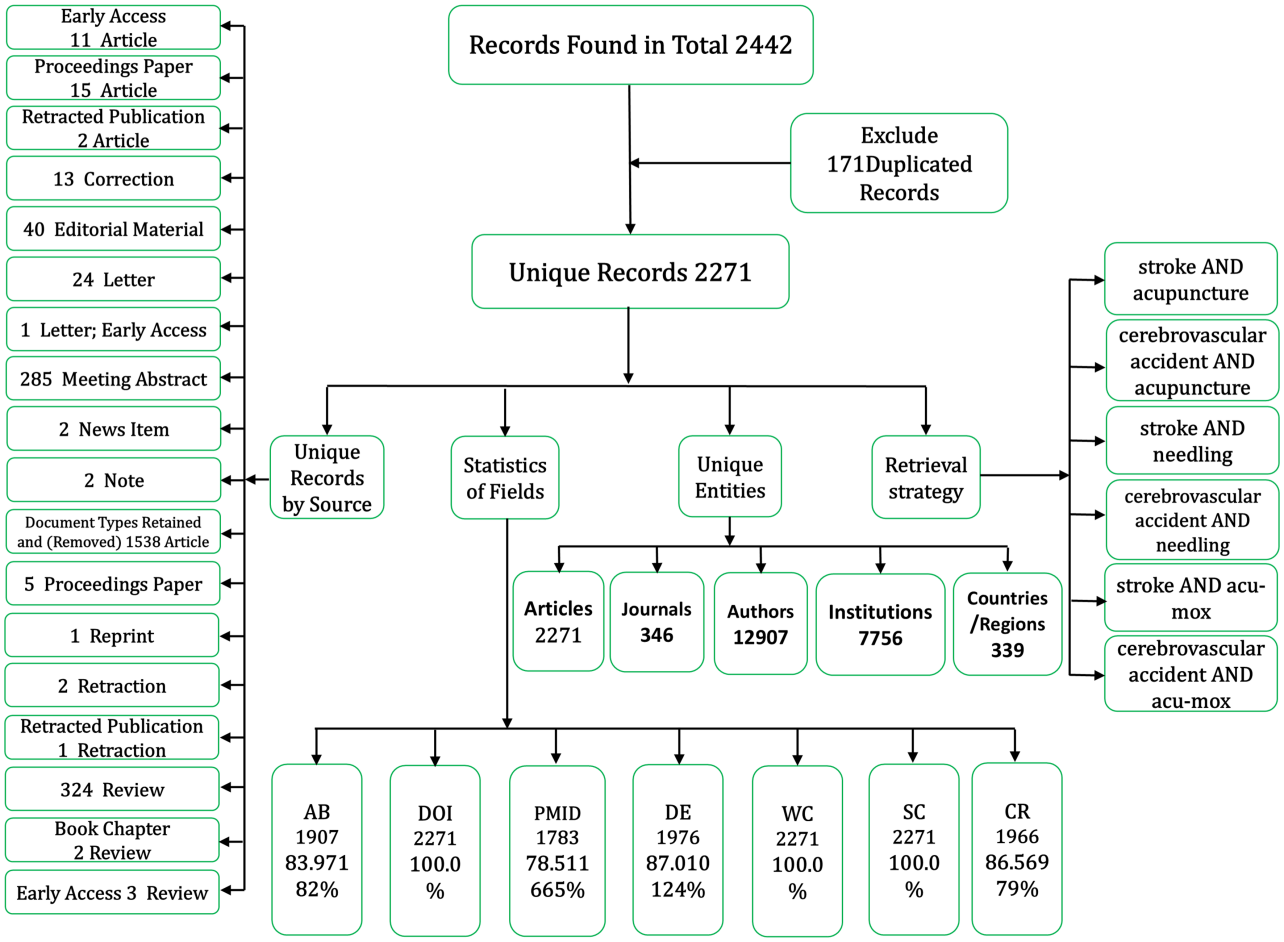
Literature screening flowchart.

The initial search of the WOS and PubMed databases identified 2,442 papers. Following data validation, deduplication, and refinement of the screening criteria, a total of 2,217 papers were deemed eligible for inclusion in the study (Figure 1).

### 3.2 Visualization of literature time and discipline

**Figure 2:**
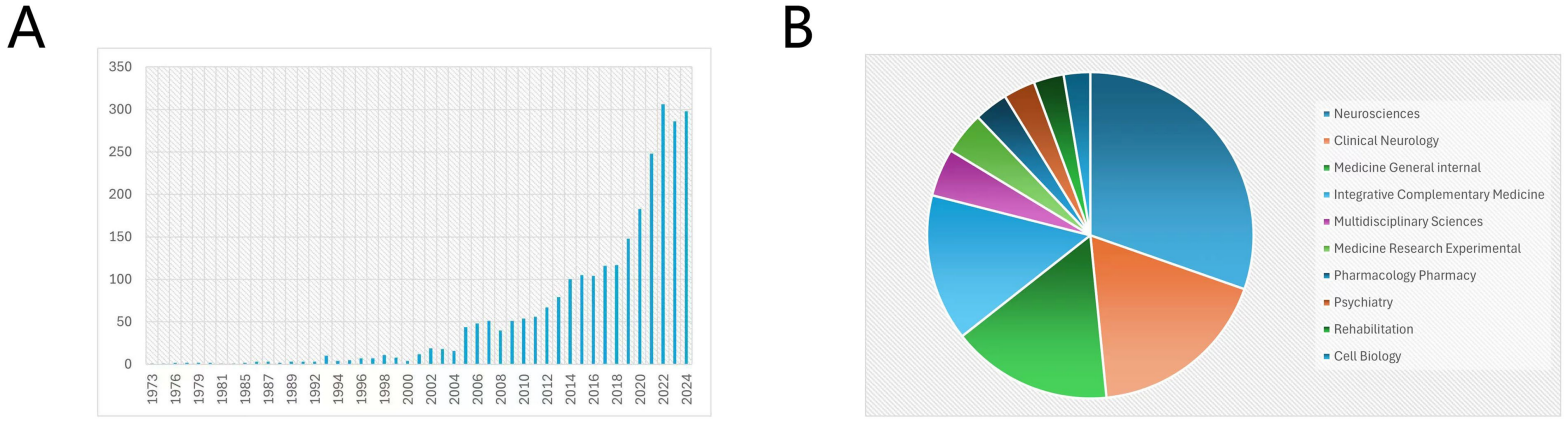
Bar chart of annual publication volume of acupuncture for stroke and pie chart of subject distribution. **(A)** Publication trends, **(B)** Subject distribution.

Acupuncture, a traditional Chinese medical practice, has been utilized in stroke treatment for centuries. However, academic research in this field began in 1973 and has experienced significant growth since 2019, indicating a surge in scholarly attention. The disciplinary distribution reveals key research hotspots, with neurosciences contributing 30.35% of the studies, clinical neurology 18.07%, and general internal medicine 15.96% (Figure 2).

### 3.3 Visualization of the country literature

**Figure 3:**
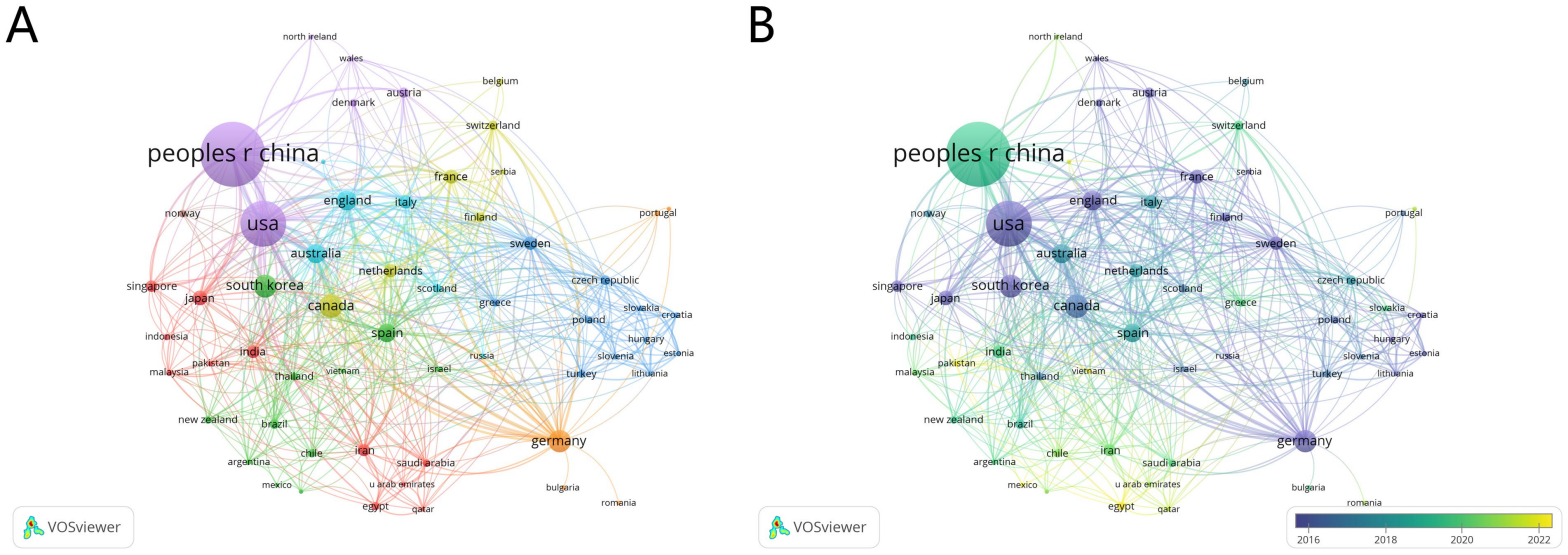
National cooperation network and geographical distribution map in the field of acupuncture for stroke. **(A)** Cluster burst, **(B)** Temporal burst.

Analysis of the WOS database identified 76 countries that have published research in this field, of which 60 were included in this study. China ranks first, contributing 876 documents with 11,024 citations and a total link strength of 155. The United States follows with 437 documents, 11,999 citations, and a total link strength of 259, while Canada ranks third with 117 documents, 3,957 citations, and a total link strength of 123 (Figure 3).

### 3.4 Visualization of institutional literature

**Figure 4:**
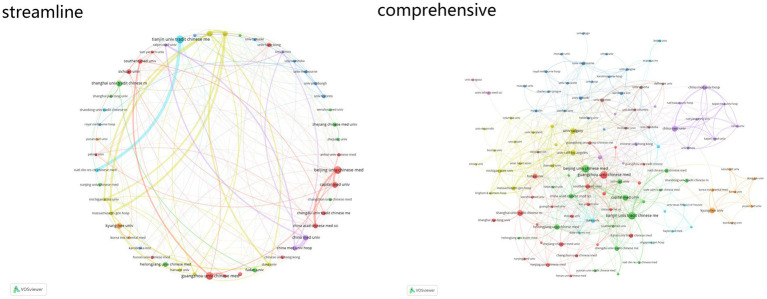
Institutional cooperation network in the field of acupuncture for stroke.

The analysis of institutional cooperation underscores key contributors to research in traditional Chinese medicine. Among 1,859 institutions with relevant publications, this study focuses on the top 40. Tianjin University of Traditional Chinese Medicine ranks first with 84 publications, 732 citations, and a total link strength of 45. Beijing University of Chinese Medicine follows with 79 publications, 1,053 citations, and a total link strength of 44. Guangzhou University of Chinese Medicine ranks third with 79 publications, 794 citations, and a total link strength of 39 (Figure 4).

### 3.5 Visualization of individual literature

**Figure 5:**
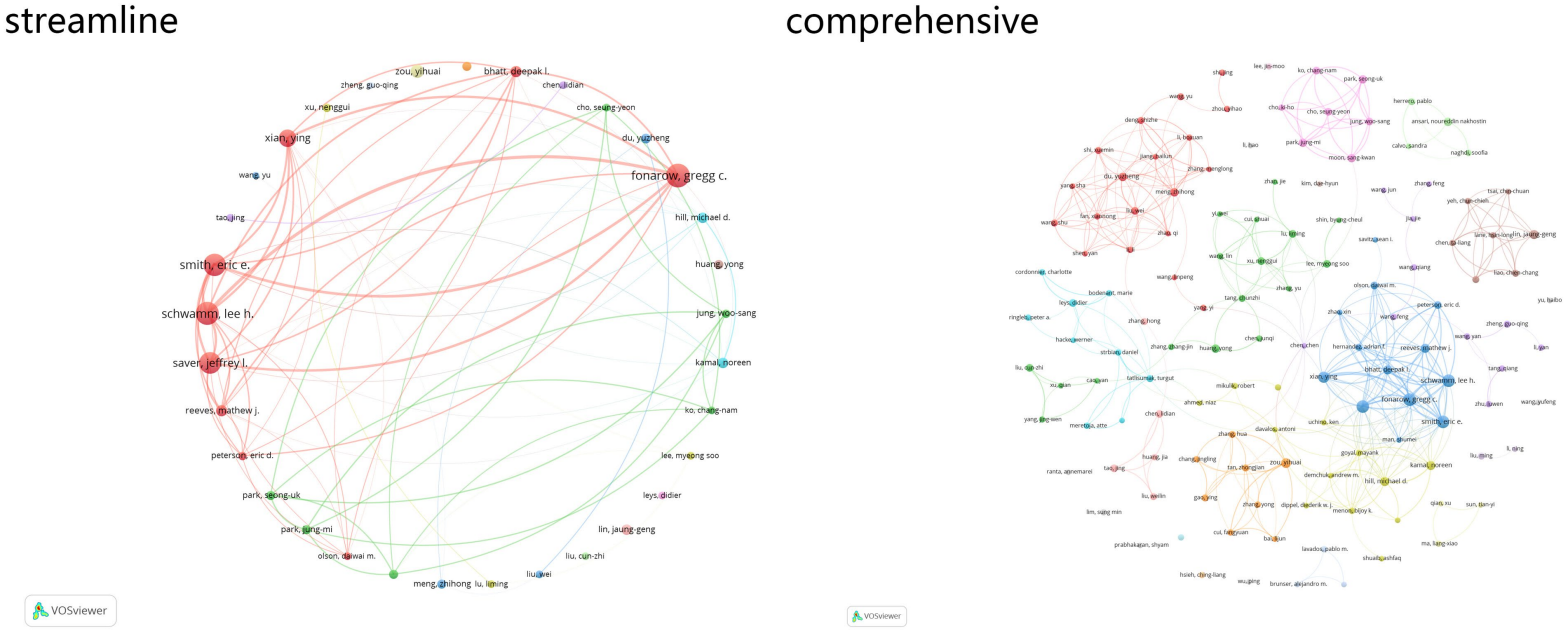
Network of authors’ collaboration in the field of acupuncture for stroke.

The analysis of individual researchers highlights the leading contributors and their collaborative networks. Fonarow Gregg C ranks first with 33 publications, 1,961 citations, and a total link strength of 149. Schwamm Lee H follows with 32 publications, 2,001 citations, and a total link strength of 145. Smith Eric E ranks third with 31 publications, 2,113 citations, and a total link strength of 141 (Figure 5).

### 3.6 Visualization of literature sources

**Figure 6:**
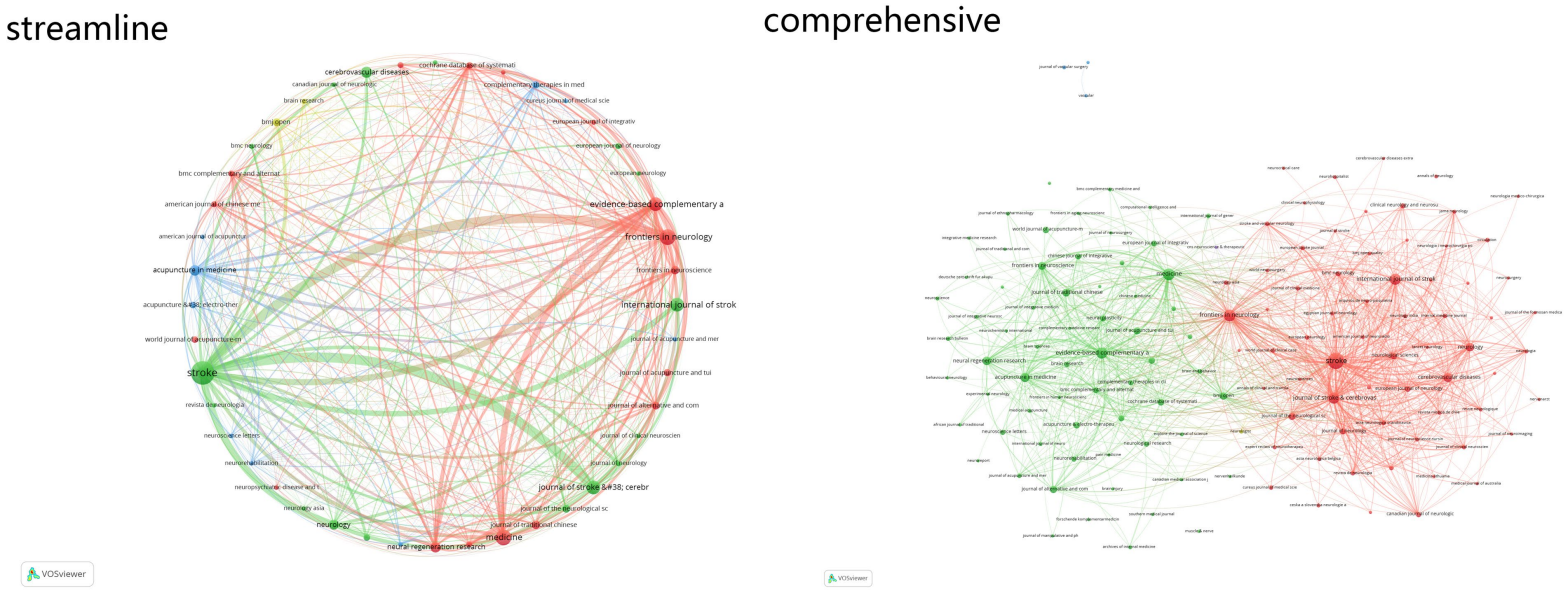
Reticular diagram of literature sources.

The analysis of literature sources highlights the academic activity and influence of key journals in the field. *Stroke* ranks first with 255 articles, 6,384 citations, and a total link strength of 760, underscoring its significant academic impact. *Frontiers in Neurology* follows with 116 articles, 664 citations, and a total link strength of 427. *Medicine* ranks third, contributing 113 articles, 481 citations, and a total link strength of 327, reflecting its central role in this research domain (Figure 6).

### 3.7 Visualization of literature citations

**Figure 7:**
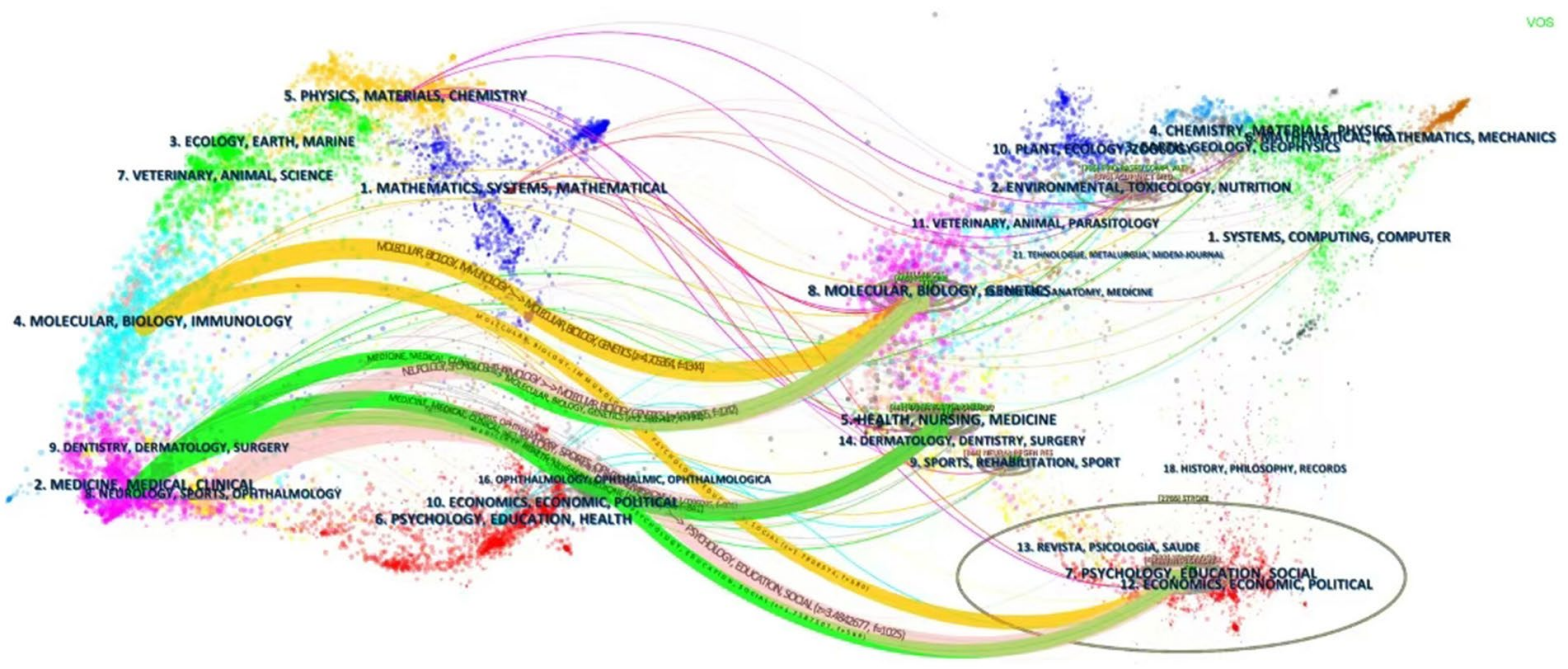
Overlay of journal double graphs.

The dual-map overlay of journals provides an intuitive depiction of differences, connections, trends, and the flow of knowledge across multiple layers of data. The analysis reveals that the citing category “Molecular, Biology, Immunology” exhibits strong citation relationships with the cited categories “Molecular, Biology, Genetics,” “Psychology, Education, Social,” and “Economics, Economic, Political.” Similarly, the citing categories “Medicine, Medical, Clinical” and “Neurology, Sports, Ophthalmology” demonstrate strong correlations with the cited categories “Psychology, Education, Social,” “Economics, Economic, Political,” “Molecular, Biology, Genetics,” and “Health, Nursing, Medicine” (Figure 7).

## 4 Visualization of literature keyword results

### 4.1 Co-occurrence of keywords

**Figure 8:**
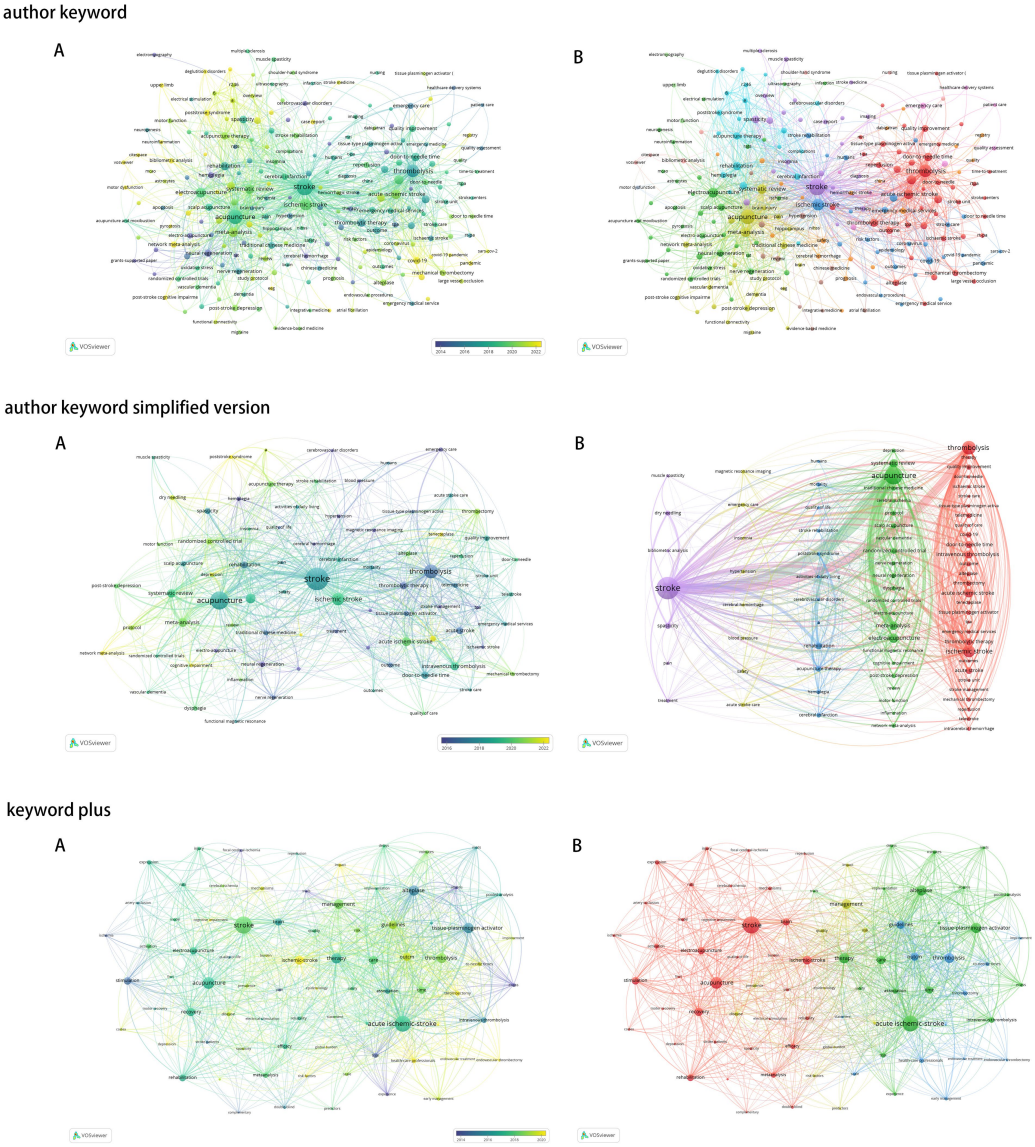
Network map and density map of keyword co-occurrence in the field of acupuncture for stroke. **(A)** Temporal burst, **(B)** Cluster burst.

Using VOSviewer to analyze the literature from the WOS and PubMed databases, the term *Stroke* appeared 630 times with a total link strength of 1,067, underscoring its pivotal role in medical research. *Acute ischemic stroke*, a critical subcategory, appeared 94 times with a link strength of 131, highlighting its importance in the field. In addition, *Acupuncture* appeared 397 times with a link strength of 666, reflecting its widespread application and influence in stroke-related research (Figure 8).

### 4.2 Prominent keywords

**Figure 9:**
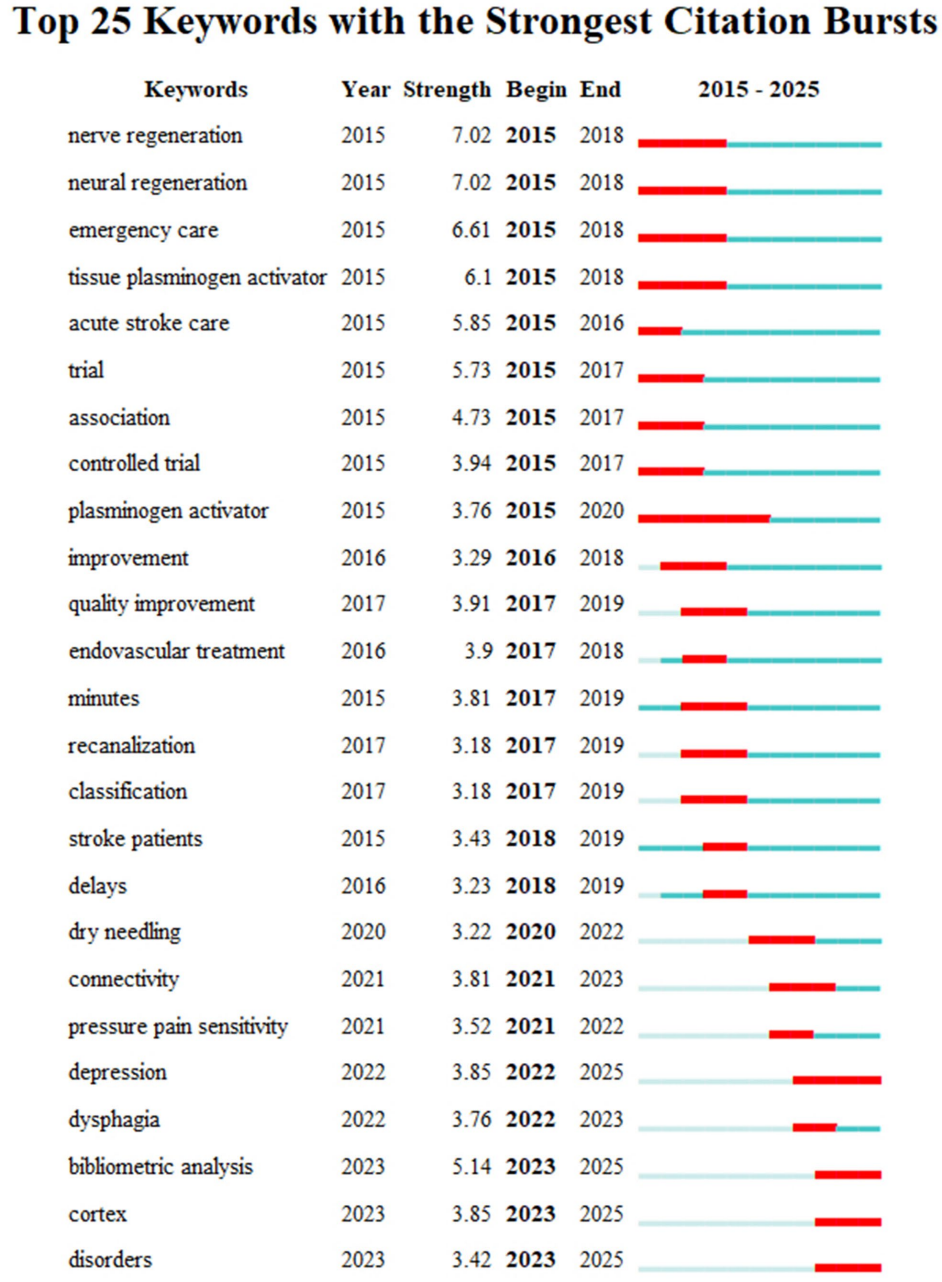
Highlights of keywords in the field of acupuncture for stroke.

Prominent keywords reflect the evolving research frontiers in stroke management. Terms such as nerve regeneration, neural regeneration, acute stroke care, and tissue plasminogen activator (tPA) highlight advancements in treatment and recovery mechanisms. Keywords such as endovascular treatment, recanalization, and minutes emphasize efficiency in acute interventions, while trial, controlled trial, and quality improvement underscore the focus on rigorous research and care standards. Patient-centered terms, including depression, dysphagia, connectivity, and pressure pain sensitivity, address post-stroke complications, while dry needling reflects interest in complementary therapies. These keywords illustrate the interdisciplinary and practical focus of stroke research, advancing both mechanistic understanding and clinical outcomes (Figure 9).

### 4.3 Keyword timeline

**Figure 10:**
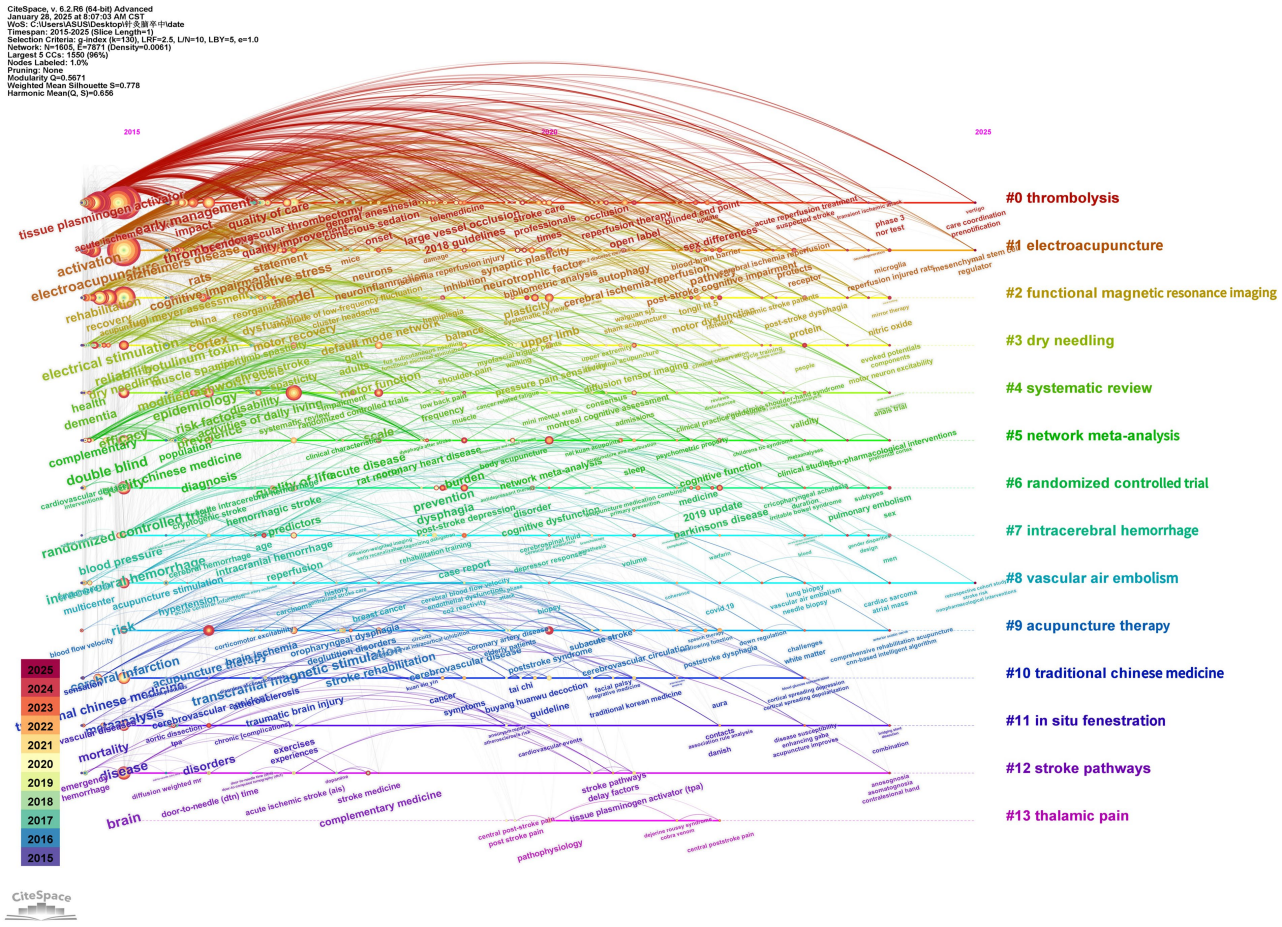
Time plot of keywords.

A timeline diagram provides a visual representation of significant events, milestones, or developmental stages in a specific field, organized chronologically. The timeline diagram in this study has a *Q*-value of 0.5671 and an *S*-value of 0.778, indicating a robust and effective analysis. The clusters identified include #0 thrombolysis and #1 electroacupuncture for stroke treatment approaches, #2 functional magnetic resonance imaging (fMRI) and #3 dry needling for diagnostic and therapeutic techniques, #4 systematic review and #5 network meta-analysis for evidence synthesis, and #6 randomized controlled trial (RCT) for methodological rigor. In addition, #7 intracerebral hemorrhage, #8 vascular air embolism, and #9 acupuncture therapy represent key areas of clinical focus, while #10 traditional Chinese medicine (TCM) and #11 *in situ* fenestration highlight innovations in treatment strategies (Figure 10).

## 5 Discussion

### 5.1 Publication volume and discipline distribution

Acupuncture, a cornerstone of traditional Chinese medicine, has been applied to stroke management for centuries (Lou and Bu, 2025). However, scientific inquiry into its mechanisms and clinical efficacy began relatively late, with the first research paper published in 1973. This delay reflects historical challenges in integrating traditional medicine into evidence-based scientific frameworks, potentially impeding earlier advancements in stroke treatment. Nonetheless, the field has seen consistent growth since 2019, culminating in a rapid increase in publications in recent years. This trend underscores the rising recognition of acupuncture as a promising therapeutic modality for stroke and highlights the urgent need for rigorous studies to validate its mechanisms and clinical efficacy (Fan et al., 2024).

From a disciplinary perspective, *Neurosciences* leads the field, accounting for 30.35% of the publications, indicating a significant focus on understanding the neurological mechanisms of acupuncture (Herrmann et al., 2025). Research in this area explores how acupuncture influences brain plasticity, neural repair, and functional recovery, offering critical insights into targeted interventions for optimizing stroke outcomes (Bae et al., 2025). *Clinical Neurology* follows with 18.07% of publications, emphasizing the translation of these mechanisms into practice. Evidence supports acupuncture’s role in improving motor recovery, reducing post-stroke complications, and enhancing patients’ quality of life. *General Internal Medicine* accounts for 15.96%, addressing essential aspects such as safety, feasibility, and cost-effectiveness, which are crucial for integrating acupuncture into clinical pathways (Gong et al., 2024).

While the dominance of neuroscience-driven research has significantly advanced understanding, it risks overshadowing other critical dimensions, such as patient-centered outcomes and long-term efficacy (Khodaie et al., 2025). Interdisciplinary approaches that incorporate public health, traditional medicine, and social sciences are necessary to evaluate acupuncture’s effectiveness holistically across diverse patient populations and healthcare systems (Li et al., 2023).

The rapid growth in publications also raises concerns about maintaining research quality. This necessitates adherence to rigorous methodological standards, including the implementation of large-scale, multicenter randomized controlled trials (RCTs) and robust control measures, to ensure reliable and reproducible findings. Establishing a robust evidence base is essential for informing clinical practice and shaping healthcare policies (Rajendran, 2024).

Acupuncture’s clinical potential lies in its role as a complementary, non-pharmacological intervention, offering benefits such as safety, minimal side effects, and accessibility. Future research should prioritize integrating acupuncture into multidisciplinary stroke care frameworks, evaluating its effectiveness in combination with pharmacological treatments, physical rehabilitation, and psychological support. In addition, cost-effectiveness analyses are needed to assess its potential to alleviate healthcare burdens (Chen et al., 2024).

As the field continues to grow, interdisciplinary collaboration, stringent research standards, and a focus on patient-centered outcomes will be critical for advancing the integration of acupuncture into stroke management. These efforts can enhance patient outcomes globally and solidify acupuncture’s position in modern stroke care (Guo and Lu, 2024).

### 5.2 Spatiotemporal distribution

Research on acupuncture for stroke treatment has demonstrated a notable trend toward internationalization, with publications spanning 76 countries. This global engagement reflects the growing recognition of acupuncture as a research focus and its potential for integration into evidence-based stroke management. China has emerged as the leading contributor, providing substantial data, theoretical frameworks, and clinical insights that serve as valuable references for researchers and practitioners worldwide. In addition, countries such as the United States and Canada have made significant contributions, showcasing robust scientific capabilities and creating opportunities for international collaboration. Such partnerships are essential for addressing challenges related to standardization and ensuring consistency in research methodologies across countries, thereby enhancing the reliability, comparability, and clinical applicability of findings (Han et al., 2024).

The analysis of institutional collaboration highlights the critical role of key institutions such as the Guangzhou University of Chinese Medicine, the Tianjin University of Traditional Chinese Medicine, and the Beijing University of Chinese Medicine in advancing acupuncture research for stroke. Guangzhou University of Chinese Medicine occupies a central position, leveraging its strong research foundation and extensive academic and clinical influence to advance understanding of acupuncture mechanisms and provide evidence for its integration into stroke care. Similarly, the Tianjin University of Traditional Chinese Medicine has made impactful contributions, with its academic influence and clinical studies strengthening the evidence base for acupuncture’s role in improving patient outcomes (Jia et al., 2024). While the Beijing University of Chinese Medicine ranks slightly below these two institutions, it maintains a high level of academic rigor and clinical relevance, contributing meaningfully to the translational potential of the field. Supporting smaller or emerging institutions alongside these leading players could foster a more inclusive research environment, enhancing the global relevance of acupuncture (Jiang et al., 2024).

At the individual level, researchers such as Zhihong Meng, Shizhe Deng, and Yuzheng Du have made significant contributions to the field. Meng Zhihong stands out with a substantial volume of high-quality publications, reflecting a rigorous research approach and extensive academic influence. His study has established a wide network of collaborations, advancing the field’s development and clinical translatability. Similarly, Deng Shizhe has produced impactful studies that provide valuable insights and data support for other scholars and practitioners. Although Du Yuzheng’s research is comparable in volume, it focuses on specific topics, offering depth and nuanced perspectives that enhance clinical understanding of acupuncture’s role in stroke recovery. Encouraging a balanced representation of researchers, including those from less prominent academic backgrounds, could broaden participation and provide a more comprehensive view of the research community and its clinical implications (Lei et al., 2024).

From a clinical perspective, the trends toward internationalization and institutional collaboration in acupuncture research have significant implications. As a non-pharmacological intervention, acupuncture offers a safe and cost-effective therapeutic option for managing post-stroke symptoms such as motor dysfunction, spasticity, and impaired quality of life. The increasing volume of research, particularly in neuroscience and clinical disciplines, has bolstered the evidence base for acupuncture’s efficacy in enhancing neuroplasticity, promoting functional recovery, and reducing complications in stroke patients. In addition, the involvement of diverse countries and institutions provides an opportunity to explore variations in acupuncture techniques, patient responses, and integration strategies, facilitating the development of tailored and effective interventions across different healthcare contexts (Li et al., 2024).

However, the rapid growth of publications raises concerns about research quality and potential regional biases, particularly given China’s dominance in publications and citations. Establishing international guidelines and collaborative frameworks is critical to addressing these issues, ensuring rigorous research standards, and fostering inclusivity across regions and institutions (Hong et al., 2024). By leveraging the strengths of international and interdisciplinary collaboration, acupuncture research can achieve greater global impact and clinical relevance. Ultimately, these efforts will benefit patients by offering a validated, accessible, and effective therapeutic option for stroke recovery and rehabilitation, further integrating acupuncture into modern medical practice (Jia et al., 2022).

### 5.3 Source and journal citation analysis

Analyzing literature sources is essential for assessing the credibility and impact of research, revealing patterns in the generation and dissemination of knowledge, and providing insights into journal activity and influence within the broader research landscape (Cao et al., 2023). In the context of acupuncture for stroke treatment, journals such as *Frontiers in Neurology* demonstrate significant academic influence by providing a high-quality platform for publishing innovative research in neurology. The journal’s focus has attracted widespread attention, accelerating advancements in this field. Conversely, the broader scope and diverse content coverage of journals such as *Medicine* may explain their relatively low focus on specialized topics such as acupuncture for stroke. This highlights the potential limitations of generalist journals in addressing niche research areas and underscores the importance of achieving a balanced representation of journals, including specialized outlets that prioritize acupuncture and stroke treatment, to facilitate in-depth exploration and focused academic discussions (Chen et al., 2020).

The activity and influence of journals in acupuncture research not only offer researchers robust platforms for disseminating knowledge but also catalyze the advancement of the field. As research in acupuncture continues to mature, it is expected that more high-impact journals will engage with this domain, contributing to the diversification of academic platforms and fostering both theoretical progress and practical applications (Chen et al., 2020). Utilizing these academic resources will enable researchers to undertake more comprehensive investigations, thereby strengthening the theoretical foundation and enhancing the clinical applicability of acupuncture in stroke treatment (Chen et al., 2016).

The journal’s dual-map overlay analysis adds further depth by visualizing citation relationships and highlighting the interdisciplinary integration of research. This approach reveals the flow and transformation of knowledge across disciplines, illustrating connections between diverse fields. For instance, in the context of acupuncture for stroke, citing categories such as *Molecular Biology, Immunology*, and *Neurology* demonstrate strong citation relationships with cited categories such as *Genetics, Psychology, Education*, and *Sociology* (Chen and Liu, 2018). This indicates that acupuncture research not only explores biological and molecular mechanisms but also intersects with the social sciences, psychology, and economics. Such interdisciplinary integration provides a multifaceted perspective on the mechanisms and clinical effects of acupuncture, enhancing both its scientific credibility and practical value (Chen et al., 2019).

In addition, citing categories such as *Medicine, Medical, Clinical*, and *Neurology, Sports*, and *Ophthalmology* are strongly correlated with cited categories such as *Psychology, Education, Sociology, Economics, Health*, and *Nursing.* This highlights the interdisciplinary nature of acupuncture research for stroke, bridging clinical studies with broader societal and healthcare-related contexts (Birch and Robinson, 2022). These intersections underscore acupuncture’s potential to address not only the biological and neurological aspects of stroke treatment but also patient-centered outcomes and social determinants of health. Recognizing these complex relationships lays the groundwork for a more holistic application of acupuncture in clinical practice, aligning research with real-world healthcare priorities (Dai et al., 2021).

In summary, the analysis of literature sources and journal dual-map overlays highlights the dynamic and interdisciplinary nature of acupuncture research for stroke treatment. The findings emphasize the critical role of specialized journals in addressing niche areas, the importance of interdisciplinary integration in advancing scientific understanding, and the value of academic platforms in fostering both theoretical innovation and clinical impact. This comprehensive approach reinforces the foundation for incorporating acupuncture into evidence-based stroke care, promoting more precise, effective, and patient-centered therapeutic strategies (Chang et al., 2018).

### 5.4 Discussion on keywords

An in-depth analysis of keyword co-occurrences reveals that *Stroke* is a core theme in medical health management research, appearing 176 times with a total link strength of 138, underscoring its central position in the field. Notably, *acute ischemic stroke*, a critical subcategory, appears 110 times with a total link strength of 201, surpassing *Stroke* itself in relevance. This highlights the significant role of acute ischemic stroke in shaping research directions and advancing stroke-related medical management. In addition, *Management* appears 92 times with a total link strength of 162, reflecting its pivotal importance across the continuum of stroke prevention, treatment, and rehabilitation. The prominence of management strategies underscores their crucial role in improving patient outcomes and elevating the overall quality of stroke health management, emphasizing the practical importance of optimizing care delivery (Chen et al., 2019).

The analysis of “prominent keywords” provides a detailed view of the dynamic evolution and emerging trends in stroke research reflecting advancements in technologies, methodologies, and clinical practices. These keywords offer critical insights into the development of the field, highlighting areas of focus across prevention, acute care, rehabilitation, and interdisciplinary integration. Terms such as nerve regeneration and neural regeneration underscore the increasing attention to cellular and molecular mechanisms underlying stroke recovery, while keywords such as acute stroke care, tissue plasminogen activator (tPA), endovascular treatment, recanalization, and minutes emphasize the critical role of timely interventions in reducing stroke-related damage (Cheng et al., 2020).

Research methodologies are reflected in terms such as trial, controlled trial, and quality improvement, underscoring the importance of rigorous evidence to inform clinical practice and refine therapeutic protocols. Patient-centered complications, including depression, dysphagia, and pressure pain sensitivity, illustrate a growing focus on improving quality of life during post-stroke recovery. At the same time, terms such as dry needling and connectivity represent innovative rehabilitation strategies, combining complementary therapies with advanced neuroimaging techniques to enhance recovery and monitor therapeutic effects.

From a broader perspective, keywords such as bibliometric analysis, classification, and stroke patients reflect the increasing emphasis on data-driven approaches and the development of standardized frameworks to analyze and address stroke-related challenges. The integration of modern technologies and traditional medical practices, exemplified by terms such as dry needling, traditional Chinese medicine (TCM), and alternative medicine, highlights the multidisciplinary and culturally diverse approaches being incorporated into stroke care (Zhu et al., 2022).

This analysis demonstrates the field’s commitment to advancing both mechanistic understanding and practical applications, ensuring that innovations in stroke management address diverse needs across prevention, acute care, and long-term rehabilitation. By bridging traditional and modern medical approaches and focusing on patient-centered outcomes, the research continues to evolve in a way that is both comprehensive and impactful (Zhu et al., 2024).

The timeline diagram offers an intuitive visualization of the field’s historical development, highlighting key milestones and trends. For example, the stroke treatment cluster (#0 thrombolysis, #1 electroacupuncture) underscores the growing recognition of both conventional and complementary therapies in stroke rehabilitation. Electroacupuncture, an advanced form of acupuncture that integrates traditional Chinese medicine principles with electrical stimulation, has gained prominence as a promising non-pharmacological intervention, reflecting the expanding role of acupuncture-based techniques in stroke recovery.

The diagnostic and therapeutic technique cluster (#2 functional magnetic resonance imaging [fMRI], #3 dry needling) highlights critical methodologies that enhance our understanding of stroke pathology and recovery mechanisms. Functional magnetic resonance imaging (fMRI) provides detailed insights into brain activity and connectivity, offering valuable information for tailoring treatment strategies. Similarly, dry needling, a therapeutic technique often used to manage musculoskeletal pain, has shown potential as an adjunctive approach to stroke rehabilitation (Wei et al., 2025).

The evidence synthesis and methodological rigor cluster (#4 systematic review, #5 network meta-analysis, #6 randomized controlled trial [RCT]) reflects the emphasis on robust research methodologies to inform clinical practice. Systematic reviews and network meta-analyses synthesize evidence across studies, establishing best practices and identifying effective interventions, while randomized controlled trials (RCTs) remain the gold standard for evaluating treatment efficacy.

The clinical focus cluster (#7 intracerebral hemorrhage, #8 vascular air embolism, #9 acupuncture therapy) highlights key areas of investigation in stroke research. Intracerebral hemorrhage and vascular air embolism represent critical conditions associated with stroke, while acupuncture therapy continues to gain recognition as a safe and effective modality for addressing post-stroke complications and enhancing recovery.

Finally, the innovation and traditional medicine cluster (#10 traditional Chinese medicine [TCM], #11 *in situ* fenestration) showcases advancements in integrating traditional and modern therapeutic approaches. Traditional Chinese medicine, including acupuncture and herbal therapies, plays a significant role in holistic stroke management, while *in situ* fenestration highlights emerging surgical techniques for addressing complex vascular conditions.

Together, these clusters emphasize the interdisciplinary and multifaceted nature of stroke research, underscoring the importance of combining evidence-based approaches with innovative and patient-centered strategies to improve outcomes in stroke rehabilitation and management (Healthcare Engineering, 2023).

In summary, the combined analysis of keyword co-occurrences, prominent keywords, timeline diagrams, and clusters reveals central themes, emerging trends, and the interdisciplinary nature of stroke research. By focusing on core topics such as acute ischemic stroke, management strategies, and therapeutic outcomes, these analyses provide a comprehensive overview of the field while addressing practical and clinical challenges. The integration of modern technologies with traditional approaches and the adoption of patient-centered strategies will be pivotal in advancing stroke research and improving outcomes for patients (Li et al., 2024).

## 6 Conclusion

This study provides a bibliometric and visualization analysis of acupuncture treatment for stroke, highlighting key research hotspots and knowledge structures within the field. As a cornerstone of traditional Chinese medicine, acupuncture has gained increasing recognition in stroke rehabilitation due to its demonstrated potential for alleviating neurological deficits, reducing complications, and improving patients’ quality of life. While these findings underscore its clinical promise, further validation through large-scale, rigorous randomized controlled trials is essential to establishing its safety and efficacy.

The integration of acupuncture with modern medical approaches offers significant opportunities but also presents challenges, particularly in the standardization of treatment protocols and addressing regional disparities in research output. Future advancements in neuroimaging, personalized medicine, and interdisciplinary collaboration hold great promise for enhancing the therapeutic potential of acupuncture. These developments are expected to provide stroke patients with more effective, evidence-based treatment options while contributing to a more comprehensive and holistic approach to stroke management.

## Data Availability

The original contributions presented in the study are included in the article/supplementary material, further inquiries can be directed to the corresponding authors.
